# In horses undergoing volatile anaesthesia, is recovery quality superior with sevoflurane compared to isoflurane?

**DOI:** 10.18849/ve.v8i1.582

**Published:** 2023-01-18

**Authors:** Alexandra Robinson+, Tsim Christopher Sun+, Eduardo Uquillas+

**Keywords:** STRESS

## Abstract

**PICO question:**

In horses undergoing volatile anaesthesia, is recovery quality superior with the use of sevoflurane compared to isoflurane during the maintenance phase?

**Clinical bottom line:**

**Category of research:**

Treatment.

**Number and type of study designs reviewed:**

Seven papers were available for critical appraisal. Of the seven papers, six were prospective, randomised trials and four of these were of crossover design. Of the same seven papers, three were experimental and four were clinical.

**Strength of evidence:**

Moderate.

**Outcomes reported:**

Five out of seven critically appraised articles found that there was no clinically significant improvement in recovery quality following volatile anaesthesia with sevoflurane compared to isoflurane. Two of the seven articles did find improvement in recovery quality following the use of sevoflurane over isoflurane, but both studies were of crossover design, one of these studies used non-blinded evaluators and the second study used both unblinded and blinded evaluators and a recovery quality scoring scale that did not show interobserver reliability.

**Conclusion:**

In healthy horses presented for elective surgical and diagnostic imaging procedures in a clinical setting, there is no significant difference in recovery quality following the use of sevoflurane or isoflurane for the maintenance phase.

## How to apply this evidence in practice

The application of evidence into practice should take into account multiple factors, not limited to: individual clinical expertise, patient’s circumstances and owners’ values, country, location or clinic where you work, the individual case in front of you, the availability of therapies and resources.

Knowledge Summaries are a resource to help reinforce or inform decision making. They do not override the responsibility or judgement of the practitioner to do what is best for the animal in their care.

## Clinical scenario

A 3-year-old Thoroughbred filly is presented to your clinic for a left carpal arthroscopy. The filly is race fit and is noted by her trainer to be highly strung. She comes with a history of having a ‘poor recovery’ following a previous anaesthetic required to facilitate the removal of an osteochondritis dissecans (OCD) lesion in a fetlock prior to beginning her race training. You are concerned about her demeanour and behaviour during the recovery phase and are interested in formulating an anaesthetic protocol for her that will minimise her fight-or-flight tendency during recovery. You have both isoflurane and sevoflurane available for use at your clinic, and you need to know if one volatile agent over the other will assist you in achieving a better recovery quality in this patient.

## The evidence

Recovery quality following general anaesthesia in horses is a multifactorial process. There were seven articles discovered that were relevant to the PICO question. There was high quality evidence in the form of three prospective, randomised, controlled clinical trials. The last four articles were of crossover design, which introduces population bias in the evaluation of recovery quality, as recovery quality has been shown to improve following successive anaesthetic episodes (Platt et al., 2018). There is no compelling evidence available in the current literature to suggest that either sevoflurane or isoflurane has any significant benefit over the other in terms of improvement of recovery quality when used in healthy patients presenting for elective procedures.

## Summary of the evidence

**Brosnan et al. (2012):** 

Population:	Healthy adult horses (research herd): Five Quarter Horses, three Thoroughbreds. Four mares, four geldings. 6 ± 2 years. 526 ± 49 kg (mean ± SD).
Sample size:	Eight horses (40 recoveries).
Intervention details:	Each horse was anaesthetised five times; at least 7 days washout period between anaesthetics. Randomly assigned a treatment order: Treatment groups differed in volatile agent used and insufflation method during recovery phase. The five treatment groups were as follows: isoflurane / 100% O_2_, isoflurane / 5% CO_2_, isoflurane / 10% CO_2_, sevoflurane / 100% O_2_, sevoflurane / 5% CO_2_. Anaesthesia: Guaifenesin 75 ± 16 mg/kg intravenous (IV) was administered prior to induction of anaesthesia with propofol 2.1 ± 4 mg/kg IV. Maintained at 1.2 minimum alveolar concentration (MAC) isoflurane (end-tidal isoflurane [EtISO] 1.57%) or sevoflurane (end-tidal sevoflurane [EtSEVO] 3.41%), as confirmed by agent monitoring, for 120 minutes in left lateral recumbency. Dobutamine was infused at a rate of 0–5.5 mcg/kg/min IV to maintain arterial blood pressure > 70 mmHg (measured invasively). Mechanical ventilation used until 20 minutes prior to recovery phase. Recovery: Recovered unassisted from left lateral recumbency. Insufflation of gases in the recovery phase consisted of 100% O_2_ or 5–10% CO_2_ in O_2_ (certified mixture), depending on treatment group, at a rate of 15 L/min. Arterial and venous blood gas and end tidal respiratory gas analysis samples drawn at 5 and 10 minutes post-disconnection. Recoveries were directly observed and evaluated for objective variables by the same investigator. Recordings of recoveries were evaluated by two independent, blinded investigators who scored the recoveries with a visual analogue scale (VAS).
Study design:	Prospective, randomised, crossover, experimental study.
Outcome Studied:	Objective: Time to first spontaneous movement (head or limbs); Time to sternal recumbency; Number of attempts to attain sternal recumbency; Time to standing; Number of attempts to attain standing position. Subjective: 100 mm VAS (combined scores from two blinded investigators); 100 mm = perfect recovery; 0 mm = catastrophic recovery.
Main Findings (relevant to PICO question):	Hypoventilation during recovery, although present in every treatment group, was significantly greater in sevoflurane anaesthetised horses. Hypercapnic hyperpnea caused a significant reduction in recovery time. Time to first purposeful movement and time to sternal recumbency were not different among treatments. Sevoflurane produced significantly longer recovery times. Strong correlation between the subjective VAS scores from the two blinded investigators (p < 0.001). No significant difference in recovery quality due to volatile agent or CO_2_ treatment existed. VAS scores (mean) differed widely between individual horses.
Limitations:	Not indicative of clinical practice – lack of typical premedication agents (α2 adrenergic agonists, phenothiazines, opioids), atypical induction regimen, no surgical stimulus, insufflation with CO_2_ not routinely practiced. Crossover design is inherently biased for evaluation of recovery quality as horses have been shown to have improved recovery qualities with successive anaesthetics. No sample size calculation. Population of horses in this study is not reflective of the breadth seen in clinical practice.

**Grosenbaugh & Muir (1998):** 

Population:	Healthy adult horses (research herd): Five grade (unregistered) Quarter Horses, two Quarter Horses, one Thoroughbred. 5–12 years. 482 ± 55 kg.
Sample size:	Eight horses (32 recoveries).
Intervention details:	Each horse was anaesthetised four times; at least 10 days washout period between anaesthetics. Treatment order randomly assigned to each horse: Concerned with the cardiopulmonary and recovery-related effects of the three volatile agents as well as two commonly used anaesthetic adjuncts (ketamine, thiopental). Four treatment groups: 1.3% halothane (group 1; 1.5 minimum alveolar concentration [MAC]), 2.0% isoflurane (group 2; 1.5 MAC), 3.4% sevoflurane (group 3; 1.5 MAC), 4.6% sevoflurane (group 4; 2 MAC). In group 1, 2 and 3, at the 30 and 60 minute mark, horses reduced to 0.5 MAC and re-anaesthetised with either ketamine (0.5 mg/kg IV) or thiopental (0.5 mg/kg IV). In group 4, the vaporiser was set to 7% and intermittent positive-pressure ventilation (IPPV) used to achieve end-tidal sevoflurane concentration (EtSevo) of 4.6% instead of using an ancillary anaesthetic agent (1.5 MAC sevoflurane administered for all but the final 30 minutes of the anaesthetic). Horses were anaesthetised for 90 minutes. Anaesthesia: Premedication: xylazine 1 mg/kg IV, guaifenesin (approximately 50 mg/kg IV) administered 10 minutes after xylazine until ataxia developed. Induction: ketamine 2.0 mg/kg IV. Horses were positioned in right lateral recumbency and breathed spontaneously (unless in group 4, in which IPPV was used to ensure horses achieved MAC sevoflurane). All volatile agents were delivered in O_2_. 16/32 (50%) of the study participants were administered xylazine 0.2 mg/kg intravenous (IV) prior to the recovery phase. Recovery: Unassisted recovery in padded stall. Video recording of recovery in its entirety.
Study design:	Prospective, randomised, crossover, experimental study.
Outcome Studied:	Objective: Time to extubation; Number of attempts to sternal recumbency; Time to sternal recumbency; Number of attempts to standing; Time to standing. Subjective: Simple descriptive scale (own manufacture).
Main Findings (relevant to PICO question):	Recovery quality from sevoflurane anaesthesia was superior to isoflurane, but similar to halothane. Time to standing in sevoflurane horses was not significantly different than that for isoflurane, but were more rapid than halothane. Times to standing appeared equalised by the administration of xylazine 0.2 mg/kg IV prior to disconnection from the circuit.
Limitations:	Horses involved in this study had a general anaesthetic prior to the study’s commencement for transposition of the left carotid artery. Crossover design is inherently biased for evaluation of recovery quality as horses have been shown to have improved recovery qualities with successive anaesthetics. No sample size calculation. Population of horses in this study is not reflective of the breadth seen in clinical practice. Used a simple descriptive scale for recovery quality scoring – this scale is insensitive, and the inclusion of objective data (time points, risk to attending personnel) is questionable. No clarification of who performed the recovery scoring in this study; assumption therefore is that the observer was not blinded, which increases risk of bias (suggest that sevoflurane anaesthesia produced a more coordinated recovery, which is entirely subjective). Recovery quality assessment was secondary to the elucidation of cardiopulmonary effects of volatile and intravenous anaesthetic agents in this study.

**Leece et al. (2008):** 

Population:	Healthy, client-owned adult horses: American Society of Anaesthesiologists (ASA) physical status classification of I–II. Undergoing anaesthesia for magnetic resonance imaging (MRI) of the distal limb. Seven Thoroughbreds, 36 Thoroughbred crosses, two Arabians, 29 Warmbloods, two ponies and one draft breed. The treatment groups were not different in age or body weight.
Sample size:	100 horses (77 recoveries; 23 were excluded due to incomplete recovery recordings).
Intervention details:	Randomisation of each horse to receive either isoflurane or sevoflurane in oxygen during maintenance phase: 38 horses received isoflurane; 39 horses received sevoflurane. Anaesthesia: Phenylbutazone 2.2 mg/kg intravenous (IV) and acepromazine 0.02 mg/kg IV administered 45 minutes prior to induction. Romifidine 0.08 mg/kg IV for premedication. Ketamine 2.2 mg/kg and diazepam 0.06 mg/kg IV for induction of anaesthesia 5 minutes after romifidine administration. All horses were maintained with intermittent positive-pressure ventilation (IPPV); initial O_2_ flow rates were 10 L/min for 10–20 minutes, then down to 5 L/min. Dobutamine was administered by constant rate infusion (CRI) to maintain mean arterial blood pressure (MAP) > 60 mmHg. Recovery: Horses were positioned to recover in the same laterality as required for imaging. Oxygen demand valve utilised until spontaneous ventilation resumed; O_2_ insufflation at 15 L/min. Xylazine 0.25 mg/kg IV used in any patient exhibiting lightness of anaesthetic plane during hoisting or positioning in the recovery box (two in each group). Recoveries were recorded from prior to placement in the recovery box until the horse was walking steadily.
Study design:	Prospective, randomised, clinical study.
Outcome Studied:	Peri-anaesthetic subjective scoring (adapted from Donaldson et al. 2000): Individual temperament; Sedation score (5 minutes post-romifidine); Induction score; Maintenance score; Depth of anaesthesia on arrival to the recovery box. Recovery objective events: Time to first movement; Time to first head lift; Time to first attempt at sternal recumbency; Time to attain sternal recumbency; Time to first attempt at standing position; Time to attain standing position; Time to full coordination (no muscle tremors or ataxia, ability to move without incoordination). Recovery subjective scoring: Recovery score – subjective scoring system (adapted from Young & Taylor, 1993); Recovery score – numerical scoring system (adapted from Donaldson et al. 2000).
Main Findings (relevant to PICO question):	No differences between treatment groups in duration of anaesthesia or times to specific recovery events. No difference in recovery scoring, number of attempts to sternal recumbency or number of attempts to standing. No difference between groups for xylazine requirements. Strong, significant correlation between the two scoring systems. Significant negative correlation between an individual horse’s pre-anaesthetic temperament was found with subjective recovery scores. Significant positive correlation between time spent in sternal recumbency and subjective recovery score. No correlation between time to standing and recovery score.
Limitations:	Acepromazine administration is associated with slow, smooth recoveries and could pose a confounding factor. Romifidine has a long half-life and the duration of anaesthesia was short, which could act as a confounding factor. Short duration of anaesthesia provides no real opportunity for volatile agent accumulation. Only one blinded observer was utilised to qualitatively score the recovery phase – this introduces bias, and may be in part why the scoring systems showed correlation. Post hoc power calculation, based on number of horses needed to detect differences in recovery time. Human error led to the loss of 23 recovery recordings.

**Matthews et al. (1998):** 

Population:	Healthy adult horses (research herd): Nine Arabians. Three mares, three geldings, three stallions. 4–20 years. 318–409 kg.
Sample size:	Nine horses (27 recoveries).
Intervention details:	Each horse anaesthetised three times; at least 6 days washout period between anaesthetics. Randomised treatment order allocation: Treatment groups were isoflurane, sevoflurane and sevoflurane with xylazine prior to recovery. Anaesthesia: Premedication: xylazine 1.1 mg/kg intravenous (IV). Induction: diazepam 0.03 mg/kg IV and ketamine 2.2 mg/kg IV. Anaesthesia was maintained for 90 minutes with the volatile agent in oxygen. Agent monitor ensured 1.2 minimum alveolar concentration (MAC). Horses positioned in right lateral recumbency. Dobutamine continuous rate infusion (CRI) to maintain mean arterial pressure (MAP) > 65 mmHg. Recovery: Xylazine 0.1 mg/kg IV given prior to recovery in sevoflurane/xylazine treatment group. Breathed room air during recovery phase. Recovery period videotaped.
Study design:	Prospective, randomised, crossover, experimental study.
Outcome Studied:	Objective: Time to first movement. Time to extubation (when horse swallowed). Time to sternal recumbency. Number of attempts to stand. Time to standing. Time to coordination. Subjective: Recovery assessment scale (simple descriptive scale that range from 1–6 with 1 being the best score) used by 3 blinded veterinarians (experienced in equine recovery). Coordination assessment scale (simple descriptive scale that ranged from 1–3 with 1 being the most coordination) used by 3 blinded veterinarians (experienced in equine recovery) to score horses at 10 minutes post-standing.
Main Findings (relevant to PICO question):	No difference between treatments for time to extubation or sternal recumbency. Mean time to first movement significantly longer in sevoflurane / xylazine treatment group. Mean time to standing significantly lower in sevoflurane only group. No difference in the mean number of attempts to stand between isoflurane and sevoflurane treatments, but the mean number of attempts to stand between isoflurane and sevoflurane / xylazine was significantly different. Recovery score means were significantly better in sevoflurane versus isoflurane treatment (P = 0.0004) and in sevoflurane / xylazine versus isoflurane treatment (P = 0.0092). Ataxia scores were significantly lower in sevoflurane versus sevoflurane / xylazine (P = 0.0001) and in sevoflurane versus isoflurane treatment (P = 0.0158). Time to safe coordination was significantly faster in sevoflurane versus isoflurane treatment (P = 0.0003) and in sevoflurane versus sevoflurane/xylazine (P = 0.0099). Time to safe coordination significantly lower in sevoflurane treated animals than in sevoflurane / xylazine or isoflurane treated animals. Individual variation in recovery score from horse to horse independent of treatment group.
Limitations:	No sample size calculation. Crossover design is inherently biased for evaluation of recovery quality as horses have been shown to have improved recovery qualities with successive anaesthetics. Scored all three anaesthetic recoveries from the same horse sequentially – removes some of the blinding and may increase the risk of bias. Unblinded reviewer’s scores were also used for statistical analysis – this introduces the unmistakable potential for risk of bias. Made own recovery scoring systems – subjective in nature and perhaps insensitive as categories show vast differences. Their recovery scoring system showed less interindividual variability in sevoflurane recoveries than in isoflurane recoveries – this finding suggests reduced reliability with their scale. Population of horses in this study is not reflective of the breadth seen in clinical practice. Absence of surgical stimulus is not indicative of clinical practice.

**Read et al. (2002):** 

Population:	Six Appaloosa / Appaloosa-Quarter horse foals: Healthy based on physical examination. Sevoflurane treatment: 92.3 ± 34.8 kg. Isoflurane treatment: 127.8 ± 22.0 kg.
Sample size:	Six foals (12 recoveries).
Intervention details:	First anaesthetic at 1 month of age; second anaesthetic at 3 months of age. Randomised treatment order allocation: Foals required surgical intervention for angular limb deformity and were scheduled for surgical procedures to occur at 1 and 3 months of age. 5/6 foals were assigned sevoflurane for their first intervention; 1/6 were assigned isoflurane for their first intervention. Anaesthesia: No premedication drugs utilised. Induction via volatile agent (5% isoflurane, 7% sevoflurane) delivered via nasotracheal tube in O_2_. Butorphanol 0.1 mg/kg intravenous (IV) administered post-induction to provide intraoperative analgesia. Mechanical ventilation used. Dorsal recumbency for surgery. Recovery: Left lateral recumbency for recovery. Room air during recovery. Recovery was assisted by two handlers blinded to treatment group. Gentle restraint in lateral recumbency until the foal deemed to be conscious and strong enough to attempt standing.
Study design:	Prospective, randomised, crossover clinical study.
Outcome Studied:	Objective: Time to first movement. Time to swallowing. Time to standing. Subjective: Simple descriptive scale (1–3) (own manufacture). Evaluated by a single observer unaware of treatment group, but involved in the hand-assisted recovery.
Main Findings (relevant to PICO question):	No difference in anaesthetic duration between groups. No difference, in any of the variables under study, were noted in the recovery phase between isoflurane and sevoflurane treated foals.
Limitations:	Crossover design is inherently biased for evaluation of recovery quality as horses have been shown to have improved recovery qualities with successive anaesthetics. Manipulation and intervention during recovery phase introduces bias, as foals not permitted to attempt sternal or standing until deemed ready for such tasks. Recovery scoring scale was simple descriptive, and perhaps over-simplified the recovery process. Only one evaluator precluded the deduction of the recovery quality scoring scale’s validity. Random assignment of treatment order saw skewed groups – differences in body weight and age as most (5/6 foals) received sevoflurane first. A foal’s tolerance to restraint would largely be a function of their familiarity with handling; bias here may be that foals presented for their second surgical procedure had already gone through hospitalisation, anaesthesia, recovery and serial bandage changes prior to presenting for their second procedure.

**Valverde et al. (2005):** 

Population:	Healthy adult horses (client owned): Physical examination and haematology used to categorise as American Society of Anaesthesiologists (ASA) physical status of I–II. All weighed > 300 kg. Surgeries lasted > 60 minutes (arthroscopies excluded).
Sample size:	54 horses (six treatment groups, nine horses per group).
Intervention details:	27 horses received isoflurane; 27 horses received sevoflurane. Random allocation to one of six groups: Treatment groups were characterised by the volatile agent used, the presence or absence of a lidocaine continuous rate infusion (CRI) and the time at which the lidocaine CRI was discontinued. Groups were: isoflurane / saline, isoflurane / lidocaine, isoflurane / lidocaine (discontinued 30 minutes prior to recovery), sevoflurane / saline, sevoflurane / lidocaine, sevoflurane / lidocaine (discontinued 30 minutes prior to recovery). Anaesthesia: Premedication: xylazine 1.0 mg/kg intravenous (IV). Induction: midazolam 0.02 mg/kg and 2.0 mg/kg ketamine IV. Intermittent positive pressure ventilation, O_2_ flow rate of 5 L/min. Maintained near 1.0 minimum alveolar concentration (MAC) for all treatment groups. Discontinued administration of volatile agent 10–20 minutes prior to recovery, continued intermittent positive-pressure ventilation (IPPV), maintained appropriate anaesthetic depth with xylazine / ketamine intermittent bolus injections. Recovery: Recovered in lateral recumbency on padded mat. Oxygen insufflation at a rate of 12 L/min during recovery. Recovery was videotaped for later individual scoring (one blinded, one not blinded to treatment group). Recovery quality scoring system used to grade horses on common behaviours exhibited during the recovery phase; this information compiled to complete a descriptive recovery score.
Study design:	Prospective, randomised, controlled clinical trial.
Outcome Studied:	Objective: Move to sternal recumbency. Time spent in sternal recumbency. Move to stand. Number of attempts to stand. Knuckling. Subjective (adapted from Donaldson et al., 2000): Overall attitude. Strength. Balance and coordination. Accident occurrence.
Main Findings (relevant to PICO question):	No effect of inhalant type of volatile (P = 0.29) or the duration of anaesthesia (P = 0.20) on the degree of ataxia. Descriptive recovery score not significantly influenced by type of inhalant (P = 0.22). No significant effect of type of inhalant on times to sternal, extubation and standing. No significant difference in the descriptive recovery scores between sevoflurane and isoflurane anaesthetised horses (when all groups considered). Horses entered the box with significantly decreased volatile concentrations (0.42 ± 0.11% isoflurane; 0.79 ± 0.20% sevoflurane).
Limitations:	Only one recovery scorer was blinded to treatment group; no difference between recovery quality score or descriptive recovery score between the two. Large number of additional anaesthetic drugs (xylazine, midazolam, ketamine, lidocaine) used during the peri-anaesthetic period. No sample size calculation.

**White et al. (2021):** 

Population:	Healthy horses (client owned): American Society of Anaesthesiologists (ASA) physical status class of I (based on thorough physical examination). Undergoing elective surgery. Older than 6 months. Had not been sedated in the 24 hours prior to anaesthesia. Groups not significantly different in age, body weight, sex, duration of surgery, duration of anaesthesia, type of surgery, position during surgery or antibiotic administration.
Sample size:	103 horses (101 recoveries).
Intervention details:	Random allocation to one of two groups: Isoflurane in oxygen for maintenance phase or sevoflurane in oxygen for maintenance phase. 49 horses received isoflurane; 52 horses received sevoflurane. Power calculation performed. Anaesthesia: Premedication: acepromazine 0.03 mg/kg and flunixin 1.1 mg/kg intravenous (IV) at least 30 minutes prior to induction; romifidine 80 mcg/kg and morphine 0.2 mg/kg IV 10 minutes prior to induction. Induction: diazepam 0.06 mg/kg and ketamine 3 mg/kg IV. Intermittent positive-pressure ventilation (IPPV) throughout. Volatile agent titrated to maintain adequate surgical depth of anaesthesia in each case (rather than chasing a certain minimum alveolar concentration (MAC) multiple). Dobutamine constant rate infusion (CRI) administered to maintain mean arterial pressure (MAP) > 60 mmHg. Ketamine or thiopental IV as rescue agents in the event of light planes of anaesthesia. Recovery: No additional use of sedatives for the recovery phase. Demand valve used until spontaneous ventilation resumed. Recoveries recorded for evaluation by two European College of Veterinary Anaesthesia and Analgesia (ECVAA) diplomats who were blinded. Two horses excluded due to incomplete recovery recordings.
Study design:	Prospective, randomised, blinded clinical study.
Outcome Studied:	Objective: Time to extubation. Time to sternal recumbency. Time to standing. Subjective: Quality of recovery scored using a previously reported system (Young & Taylor, 1993), where 0 = catastrophic recovery and 5 = perfect recovery.
Main Findings (relevant to PICO question):	No significant difference in: MAC hours, hypotensive indexes, MAC multiple at disconnection. Median time to extubation was the same, but the range was significantly different. Time to sternal recumbency was significantly longer in sevoflurane than isoflurane horses (P = 0.03). No difference in time taken to stand or the number of attempts to stand between groups. No difference in recovery score between isoflurane and sevoflurane horses between the blinded evaluators or the attending anaesthetist although the attending anaesthetist did assign higher values to the sevoflurane recoveries.
Limitations:	Acepromazine administration is associated with slow, smooth recoveries and could be a confounding factor. Romifidine has a long half-life and the duration of anaesthesia was short, which could act as a confounding factor. Using anaesthetic-related data from four different anaesthetists can introduce variability, but the anaesthetic regimen was protocolised which may act as a control for this variation.

## Appraisal, application and reflection

Equine anaesthesia is associated with an overall mortality rate of 1.0% (Gozalo-Marcilla et al., 2021) which is a modest improvement from mortality rates of 1.9% reported nearly 20 years ago (Johnston et al., 2002). Certain patient demographics are at higher risk of mortality than others, such as those with high American Society of Anaesthesiologists (ASA) physical status, extremes of age, body weight, fracture reparation, emergency laparotomy, increased anaesthetic time, procedures occurring out of hours, patients induced without premedication and maintenance with volatile agents (Johnston et al., 2002; and Johnston et al., 1995). Causes of anaesthetic-related mortality are variable and can include intraoperative cardiac arrest, fractures, luxations, neuropathy, myopathy, spinal cord malacia and respiratory obstruction (Dugdale & Taylor, 2016). Recovery from general anaesthesia has long been incriminated as the most dangerous part of the perianaesthetic period for equine patients, with 92% of overall morbidity and mortality occurring within this period (Laurenza et al., 2020). As such, the elucidation of specific anaesthesia-related factors for the improvement of recovery quality has been of considerable interest.

A review of the literature was carried out to answer the PICO question, followed by an article exclusion process resulting in seven peer-reviewed publications appropriate for critical appraisal. All seven articles have clear relevance to the PICO question. Of the seven articles, all are prospective, randomised trials. Four of these were crossover in design. There was, at least partial, blinding of recovery evaluators to treatment group in six of the articles. Four of the articles used client-owned horses, whereas the other three used research herds. The clinical trials, of which there were three, were most reflective of current standards in equine clinical anaesthesia. The articles retrieved were relatively recent, spanning only the last two decades.

In several large-scale multi- and single-centered epidemiological morbidity and mortality studies, volatile anaesthetic agents have been incriminated as a risk factor for mortality (Bidwell et al., 2007; Dugdale et al., 2016; and Johnston et al., 2002). The volatile anaesthetic agents are well known for their dose-dependent cardiorespiratory depression (Grosenbaugh & Muir, 1998). Such depression can lead to deleterious clinical sequelae such as hypotension, hypoventilation, hypercapnia and hypoxemia. Although direct causal relationships have not been identified, the presence of hypotension and hypoxemia have deleterious effects on muscle perfusion and tissue oxygenation, which could negatively impact recovery quality. Despite this, volatile agents are unlikely to be abandoned in equine anaesthesia due to their use during long and invasive procedures, minimal metabolism, ventilation-dependent elimination, titratability, and the ease with which their concentrations are monitored.

This critical appraisal was limited to sevoflurane and isoflurane, as they are the predominant volatile agents currently in use (Gozalo-Marcilla et al., 2021). Sevoflurane has been proposed to produce superior recovery quality in equine patients compared to isoflurane (Grosenbaugh & Muir, 1998). The pharmacokinetic and pharmacodynamic profile of sevoflurane suggests that it should have clinical advantages over isoflurane (Steffey, 2002). Sevoflurane has a lower blood / gas partition coefficient, indicating lower solubility of the agent in the blood. This results in a more rapid equilibration of partial pressure between the alveolar space and the blood and brain, leading to more rapid induction and recovery phases. This may increase the speed in which anaesthetic depth can be changed through titration of volatile agent delivery. Sevoflurane accumulated in the adipose tissue during long periods of general anaesthesia is also more rapidly eliminated than isoflurane, which may be an important factor during the recovery phase where horses can experience emergence delirium and dysphoria due to the continued presence of a volatile agent in their system. It may be that the advantage in using sevoflurane over isoflurane may only become evident after long (>3 hour) duration anaesthetics, although there are no comparative studies available that have investigated this.

Recoveries of good quality are characterised by the absence of emergence delirium, dysphoria or ataxia and the presence of adequate musculoskeletal strength. These characteristics lead to balanced and coordinated recoveries, reduced knuckling and falling events with fewer attempts to sternal recumbency and standing. Recoveries with these characteristics are likely to be qualitatively calm and smooth, reducing the opportunity for self-inflicted injury. Horses are prone to displaying fight-or-flight responses during emergence from general anaesthesia, and it has been postulated that the individual horse’s temperament may influence recovery (Brosnan et al., 2012; Leece et al., 2008; and Matthews et al., 1998). In addition, time spent in lateral recumbency, number of attempts to sternal recumbency, time spent in sternal recumbency, number of attempts to standing and time to standing are common objective variables used to characterise the recovery phase.

The assessment of recovery quality following use of a specific volatile agent is challenging. The use of ancillary anaesthetic agents is necessitated as part of a balanced anaesthetic technique and may include the use of injectable drugs for premedication, induction, rescue anaesthesia and the use of additional sedative and analgesic drugs in the recovery phase. The effects of ancillary agents in the recovery phase can be difficult to quantify, especially where long-lasting agents, such as acepromazine are involved in the anaesthetic protocol (Knych et al., 2018). Recovery quality will most likely be attributable to the volatile agent if the use of other ancillary agents within the protocol is standardised. Furthermore, it has become standard practice to administer additional sedative drugs during the recovery phase to improve recovery quality by decreasing opportunity for disorientation and volatile agent-related dysphoria (Santos et al., 2003; and White et al., 2021). Leece et al. (2008) and White et al. (2021) published results from prospective clinical trials using stringent anaesthesia protocolisation devoid of recovery sedatives. The results from each study concluded that there was no significant difference in recovery quality following sevoflurane versus isoflurane anaesthesia. There were no articles available for appraisal that evaluated volatile agents without the use of ancillary drugs.

The scoring of recovery quality following general anaesthesia is often subjectively assessed and qualitative in nature. Vettorato et al. (2010) investigated the reliability of four distinct recovery quality scoring systems (RQSS) through use of two groups of evaluators, 117 final-year veterinary students and 12 experienced equine anaesthetists. The four RQSS evaluated were a visual analogue scale originally reported by Hubbell in 1999, a composite scoring system (Donaldson et al., 2000), a simple descriptive scale (Young & Taylor, 1993) and the Edinburgh scoring system (Vettorato et al., 2010). Results revealed that the four RQSS exhibited moderate-high reliability, suggesting that there was significant interobserver agreement amongst scores given by blinded evaluators and that the scales were reliable even when used by inexperienced operators. Ideally, high interobserver agreement of recovery scales should be identified to reduce bias and enable meaningful comparisons between studies. Although a perfect RQSS has not yet been developed, recovery scales should be objective, sensitive in detecting recovery quality differences and be adapted to accommodate the conditions of the study facility (Valverde et al., 2005). In addition to this, researchers should seek to limit bias by blinding recovery evaluators to treatment groups.

Of the seven studies evaluated, four utilised RQSS that were recognised as being reliable by Vettorato et al. (2010). Valverde et al. (2005) utilised a modified composite scoring system, Leece et al. (2008) utilised a modified combination of a composite scoring system and simple descriptive scale, Brosnan et al. (2012) utilised a visual analogue scale and White et al. (2021) utilised a simple descriptive scale. Each of these scales had roots in the published grandfather articles referenced in Vettorato et al. (2010). All four articles used recovery evaluators that were blinded to treatment group, thus minimising the possibility of bias. Valverde et al. (2005) even proved the reliability of their modified composite scoring system by having one blinded and one unblinded evaluator to score recoveries. None of the RQSS used in these studies detected significant differences in recovery quality between sevoflurane and isoflurane recoveries. The remaining three articles used simple descriptive scales of their own manufacture that resulted in discrepant results (Matthews et al., 1998), had non-existent blinding processes (Grosenbaugh & Muir, 1998), and utilised hand-assisted recovery techniques (Read et al., 2002), thus increasing the potential for bias and possibly misleading results.

A recent publication by Platt et al. (2018) showed that habituation and learning during the recovery phase following sequential general anaesthetic episodes in equine patients occurs and culminates in improvement of recovery quality. This phenomenon may reduce the reliability of crossover designed research studies when determining recovery quality in horses. Four of the appraised articles were crossover designs. Interestingly, there were contrasting results. Two studies reported no difference in recovery quality when using sevoflurane versus isoflurane (Brosnan et al., 2012; and Read et al., 2002), while the other two suggested that sevoflurane recovery quality was superior (Grosenbaugh & Muir, 1998; and Matthews et al., 1998). Study participants were anaesthetised on as little as two (Read et al., 2002) to as many as five (Brosnan et al., 2012) separate occasions for study purposes. The crossover design and ample opportunity for learned behaviour development make the results of these articles difficult to interpret. Three of the four crossover designed studies had additional significant limitations that clouded their clinical relevance, such as unblinded recovery evaluators (Grosenbaugh & Muir, 1998), an insensitive and unreliable RQSS that may have precluded detection of specific aspects of recovery behaviour (Matthews et al., 1998) and hand-assisted recovery techniques (Read et al., 2002).

The use of anaesthesia-naïve animals may be the most logical population in which to study recovery quality. Of the articles appraised, three were clinical trials in which anaesthesia-naïve animals were enrolled (Leece et al., 2008; Valverde et al., 2005; and White et al., 2021). These three studies also had the strongest study designs, used reliable RQSS, had large sample sizes, reflected the general horse population and used anaesthetic protocols common in clinical practice. In addition to this, both Valverde et al. (2005) and White et al. (2021) cases were surgical procedures and those in Leece et al. (2008) involved diagnostic imaging, both scenarios of which are relevant to clinical practice. Furthermore, the studies produced by Leece et al. (2008) and White et al. (2021) were only concerned with recovery quality differences, thus making their results highly relevant to our analysis. For these reasons, these three articles likely offer the most accurate and unbiased information available to answer the PICO question. None of these studies detected any significant difference in recovery quality following sevoflurane versus isoflurane anaesthesia.

The finding from this critical appraisal was supported by the findings of a recent systematic review. Loomes & Louro (2021) deduced that there is no conclusive evidence that any given volatile agent is superior to another in terms of recovery quality. Loomes & Louro (2021) recognised that there is a relative dearth of literature on the topic, but the articles available typically have strong study designs with blinded recovery quality evaluators and the use of a validated RQSS. Limitations in the articles were the inconsistent use of sedatives prior to the recovery phase, general lack of power calculations, although Leece et al. (2008) did perform a post hoc power, and the presence of multiple study objectives. These limitations were also present in the currently appraised articles, and they seem to be common limitations in articles involving equine anaesthesia in general. Despite these limitations, Loomes & Louro (2021) suggest that there is moderate evidence to support their conclusion. A recently published expert opinion (Bettschart-Wolfensberger, 2021) combining extensive clinical experience and an independent dissection of the literature has culminated in the same conclusion.

After complete appraisal of the evidence, the authors conclude that there is moderate evidence in the literature to support that there is no clinical difference in recovery quality following sevoflurane versus isoflurane anaesthesia in healthy horses under clinical and experimental conditions. Sevoflurane and isoflurane both provide rapid, smooth recoveries of good quality. In the absence of clear and causal relationships between specific volatile anaesthetic agents and anaesthetic outcome, an individual clinician’s choice of volatile agent should be based on user familiarity, relative anaesthetic risk of the patient, anticipated duration of anaesthesia and the potential environmental impacts. Continued research into the benefits of administering additional sedative drugs in attempts to improve recovery quality are required. Further evaluation of volatile agent influence on recovery quality may require the use of sick horses and long (> 3 hour) durations of anaesthesia.

## Methodology

**Search Strategy:** 

Databases searched and dates covered:	CAB Abstracts on Web of Science Platform: 1910–2022 PubMed accessed via NCBI: 1950–2022
Search strategy:	CAB Abstracts: TS=(horse* OR equus OR equin* OR equid* OR mare OR mares OR gelding* OR stallion* OR pony OR ponies OR broodmare* OR foal* OR colt* OR filly OR fillies) TS=((volatile* or inhalation*)NEAR/2(anaesthe* or anesthe* or agent*)) TS=(isoflurane) TS=(sevoflurane) #1 AND (#2 OR (#3 and #4)) PubMed: “Horses” [Mesh:NoExp] Horse* OR equus OR equin* OR equid* OR mare OR mares OR gelding* OR stallion* OR pony OR ponies OR broodmare* OR foal* OR colt* OR filly OR fillies “Anaesthesics, Inhalation” [Mesh:NoExp] “volatile anaesthetic” OR “volatile anaesthetics” OR “volatile anaesthesia” OR “volatile anesthetic” OR “volatile anesthetics” OR “volatile anesthesia” OR “inhalation anaesthetic” OR “inhalation anaesthetics” OR “inhalation anaesthesia” OR “inhalation anesthetic” OR “inhalation anesthetics” OR “inhalation anesthesia” OR “volatile agent” OR “volatile agents” OR “inhalation agent” OR “inhalation agents” Isoflurane Sevoflurane (#1 OR #2) AND (#3 OR #4 OR (#5 AND #6))
Dates searches performed:	26 May 2022

**Exclusion / Inclusion Criteria:** 

Exclusion:	Articles irrelevant to PICO question. Expert opinion. Not available in English. Systematic review. Duplicates.
Inclusion:	Relevant to PICO question, peer-reviewed and available in English.

**Search Outcome:** 

Database	Number of results	Excluded – Irrelevant	Excluded – Expert opinion	Excluded – Not in English	Excluded – Systematic review	Total relevant papers
CAB Abstracts	877	867	1	1	1	7
PubMed	536	529	0	0	1	6
Total relevant papers when duplicates removed						7

### Acknowledgements

The authors wish to acknowledge the assistance of Ms Monica Cooper in the process of formulating a literature search strategy for this review.

### ORCID

Alexandra Robinson: https://orcid.org/0000-0003-4937-9552 Tsim Christopher Sun: https://orcid.org/0000-0003-1033-8251 Eduardo Uquillas: https://orcid.org/0000-0002-4227-2173

### Conflict of Interest

The authors declare no conflicts of interest.
